# Notes and News

**Published:** 1900-07-21

**Authors:** 


### Notes and News.

HOSPITAL MEETING.

Hospital for Epilepsy and Paralysis.—Annual Meeting at three p.m., on Wednesday, July 25th.

A telegram which reached London last Friday states that the Imperial Yeomanry Hospital at Deelfontein has now upwards of 1,000 patients in it.

The Shah of Persia has been taking the waters at Contrexeville, and before he left he gave expression to the gratification which his visit had afforded him.

The treasurer of Queen's Hospital, Birmingham, has received a cheque for £1,250 from Mrs. Poncia, of Edgbaston, for the endowment of a bed in memory of her husband, the late Mr. John Poncia.

An infectious diseases hospital is in course of erection for the Fylde Joint District near Lytham. It is to consist of two pavilions, for typhoid and scarlet fever respectively, with the usual offices and buildings.

According to a statement made by Mr. Wyndham, it appears that the number of hospital orderlies employed in South Africa on May 19th was 3,943, including 986 St. John Ambulance Brigade, 91 Militia Medical Staff Corps, and 492 volunteers. This does not include the staff of such hospitals as the Yeomanry, the Portland, the Langman, &c., the subordinate male staff of which numbers 519. It is not clear, however, how many of this total are employed exclusively in the hospitals.

A Bill to amend the Medical Acts, 1858 to 1886, was introduced in the House of Commons last Friday by Sir Richard Jebb. It has been framed by the General Medical Council, after communication with the Universities and other licensing bodies. Clause 1 enables the Council to erase from the register for a limited period the name of a practitioner who has been guilty of misconduct, thus giving the Council the power, of which it has sadly felt the want, of being able to graduate the penalty according to the offence. Clause 2 enables the Council to withdraw his diploma from a person whose name has been erased from the register. Clause 3 provides that all fines and penalties recoverable under the Medical Acts shall be paid to the General Medical Council, to be applied in aid of the expenses of executing these Acts.

At the age of 75 Professor Potain has retired from the Chair of Clinical Medicine. On July 3rd he gave his last lecture in the theatre of the Hôpital de la Charité, on which occasion a large number of leaders in medicine, his former pupils, assembled to bid him farewell and to show their sympathy and affection for one who for 40 years had worked and taught in the wards of the hospital.

According to the observations taken by Mr. Sowerby Wallis, the shade temperature in London on July 16th rose to 95·2 deg., the highest point reached since observations were commenced at Camden Square by the late G. J. Symons in the year 1848. During the 42 years the temperature has reached 92 deg. on only 12 occasions, six of which have been in July, five in August, and one in June. The highest record in any year till last Monday was on July 15th, 1881, when it reached 94·6. All these observations were taken on a Glaisher stand.

The plague seems to be gradually subsiding in Australia. A Reuter telegram, dated July 14th, reports two more cases of plague in Sydney, where, as in other places, the outbreak has shown a tendency to locate itself in the Chinese quarter. Dr. George Shrady, of New York, recently visited the Chinese quarter of San Francisco to report upon the presence of plague amongst the inhabitants. His investigations are likely to be followed by a complete cleansing of one of the most filthy neighbourhoods known. In San Francisco this same tendency of the disease to infest the parts of the town inhabited by the yellow race has led to difficulties between the authorities and the Chinese; the latter having entered a suit to test the legality of the differential treatment meted out to them. Much difficulty sometimes also arises from the fact that on the subsidence of an outbreak of plague it is by no means easy to say how soon it is justifiable to relax precautionary measures. The last news from Hong Kong is more serious, 58 deaths from plague having been reported during last week.

Among those who are supposed to have fallen in the terrible massacre in Peking we notice the names of several members of the medical profession. Dr. Wordsworth Poole, physician to the Embassy, was a member of the Royal College of Physicians of London. He studied at Cambridge and at Guy's. It is understood that his wife was with him in Peking. Dr. Coltman, an American, was professor at the Chinese University. Miss Lillie E. V. Saville, M.D.Brux., qualified in 1894, having studied at the London School of Medicine for Women, and went out as a medical missionary in 1895. She was the daughter of the Rev. A. T. Saville, of Rye, Sussex, who himself was formerly one of the missionaries of the London Missionary Society. The Rev. Thomas Biggin, M.A., although not qualified, had been a medical student. While at Oxford he had commenced the full course for the medical degree, intending to offer himself as a medical missionary, but he ultimately decided to become an educational missionary, and went out to China last year. Lastly we have to add to the sad roll the name of Dr. G. E. Morrison, the special correspondent of the *Times* in Peking, who was a graduate of Edinburgh, where he qualified in 1887.

